# The Dilemma Behind Negative Troponin: A Case Report

**DOI:** 10.7759/cureus.34377

**Published:** 2023-01-30

**Authors:** Nava R Sharma, Bharosa Sharma, Madalasa Pokhrel, Sudarshan Gautam, Saral Lamichhane

**Affiliations:** 1 Medicine, Manipal College of Medical Science, Pokhara, NPL; 2 Internal Medicine, John H. Stroger Jr. Hospital of Cook County, Chicago, USA; 3 Internal Medicine, Montefiore Medical Center, New Rochelle, USA; 4 Internal Medicine, Maimonides Medical Center, Brooklyn, USA; 5 Department of Internal Medicine, Gandaki Medical College, Pokhara, NPL

**Keywords:** ventricular thrombus, acute coronary syndrome (acs) and stemi, st-elevation myocardial infarction (stemi), false negative troponin, cardiac troponin

## Abstract

Acute coronary syndrome remains the primary cause of mortality and morbidity in the United States. Cardiac ischemia is a consequence of an imbalance between oxygen demand and supply. The sensitivity of troponin is above 99% in diagnosing cardiac injury; rare exceptions can occur, however. We present a case of acute coronary syndrome with a negative troponin level, even on repeated testing using different methods at two different centers.

## Introduction

Acute coronary syndrome includes ST-elevation myocardial infarction (STEMI), non-ST elevation myocardial infarction (NSTEMI), and unstable angina. Acute coronary syndrome can present as a STEMI when a thrombus completely occludes an epicardial coronary artery [1]. Diagnosis of STEMI is based on clinical characteristics and persistently elevated ST segment elevation in electrocardiography (EKG) [2]. There may be normal troponin during the initial presentation in patients undergoing immediate coronary intervention [2,3].

We are presenting here a unique and rare case of a 60-year-old man who had acute coronary syndrome despite having persistently normal cardiac injury markers.

This article was presented as a poster at the ACC 2022 Annual Scientific Session in Washington, DC, USA, on April 02, 2022.

## Case presentation

A 60-year-old man with no known medical history presented to the emergency room with chest pain for two days. The pain had started while he was sitting. It was located substernal and was crushing in nature, with a severe intensity that he rated as ten out of ten. He took Ibuprofen, which provided some relief. The pain was aggravated by exertion and coughing, prompting him to come to the hospital. He had a similar history of left-sided chest pain one month ago, which was relieved on its own. The pain was mild and lasted for a few minutes. He was an active smoker with a history of smoking five cigarettes daily for the last 40 years.

**Figure 1:**
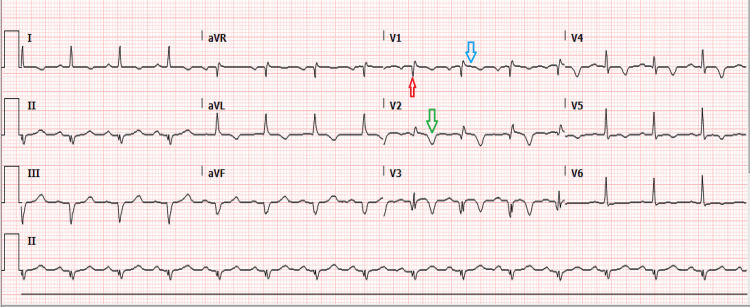
Initial EKG showing Q wave (red arrow), ST-elevation (blue arrow), and T wave inversion (green arrow) in the anterolateral leads.

On examination, he had a blood pressure of 100/60 mm Hg, a heart rate of 109 beats per minute, a respiratory rate of 22 breaths per minute, and an oxygen saturation of 94% in room air. Systemic examination did not show any significant findings. The initial EKG showed Q waves, ST-segment elevation (1 mm), and T-wave inversion in the anterolateral leads (Figure 1). His troponin-I and troponin-T remained normal throughout the hospital stay, even on repeated testing in our and other centers.

His other test reports at the time of presentation are shown in Table 1.

**Table 1:** Laboratory results at the time of presentation.

Investigations	Values	Normal value
White blood count	10.8	3.5–11 × 109/L
Hemoglobin	15.7	12–18 g/dl
Platelets	259	140–400 × 109/L
Alkaline phosphatase level	83	0–129 unit/L
Total bilirubin	5.4	0.3–1.0 mg/dl
Aspartate aminotransferase level	25	10–35 unit/L
Alanine aminotransferase level	17	10–35 unit/L
Lactate dehydrogenase	196	110–270 U/L
Sodium	132	135–145 mM/L
Potassium	4.0	3.5–5.1 mM/L
Chloride	99	98–112 mM/L
Blood urea nitrogen	10	12.0–20.0 mg/dl
Creatinine	1.1	0.50–1.50 mg/dl
Total cholesterol	108	<200 mg/dl
Troponin I	<0.019	0.02–0.05 ng/mL
Creatine kinase-muscle/brain (CK-MB)	Negative	Negative
Heterophile antibody	Negative	Negative

With thrombolysis in myocardial infarction (TIMI) 1 flow, left heart catheterization revealed 99% proximal left anterior descending (LAD) occlusion. Mild obstruction was also seen in the left circumflex and right coronary arteries. The LAD lesion was stented using a 3.0 mm × 38 mm drug-eluting stent (DES). After stent deployment, a thrombus was noted proximal to the stented segment. We deployed a 3.5 mm × 15 mm drug-eluting stent in the ostial LAD.

**Figure 2:**
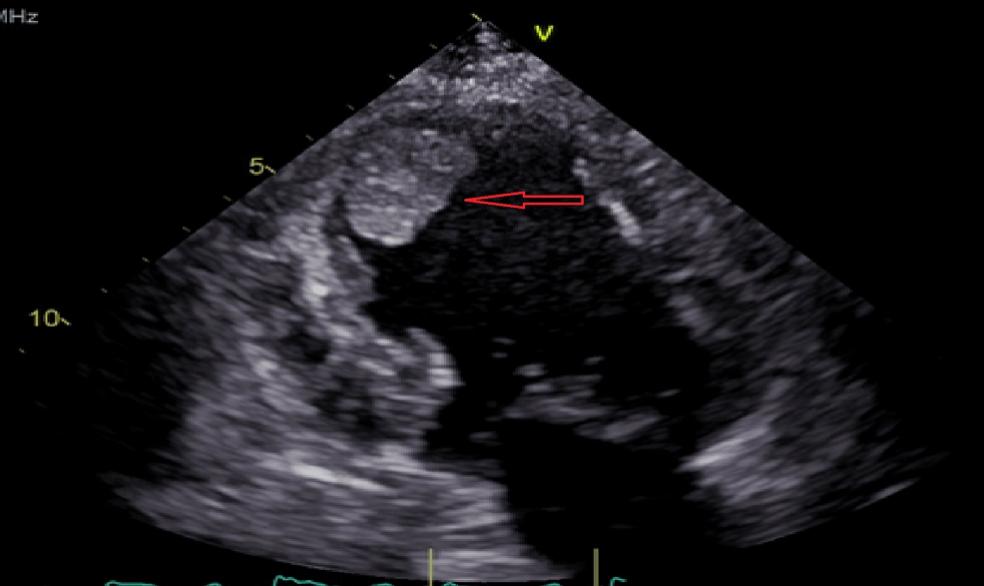
Echocardiography showing thrombus in the left ventricle.

The echocardiography showed moderately decreased left ventricular function with an ejection fraction of 30-35%. There was a fixed spherical apical thrombus with a dimension of 2.7 cm × 2.3 cm, as shown in Figure 2. There was akinesis of the apical, anterior, mid-anteroseptal, mid-apical septal, apical-lateral, and apical myocardium. In addition, there was severe hypokinesis of the basal mid-anterior and mid-anterolateral myocardium.

**Figure 3:**
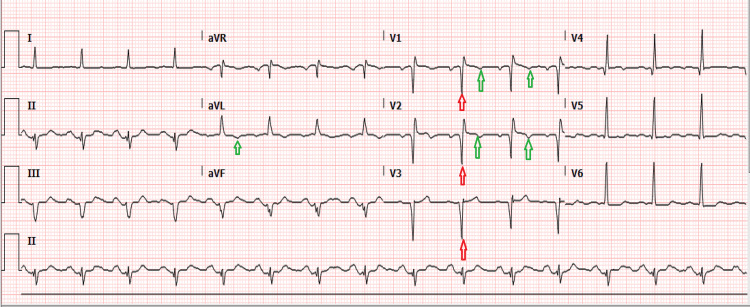
EKG during follow-up showing pathological Q waves (red arrow) and T-wave inversions (green arrow) in anterolateral leads.

The aortic root measured 4.1 cm at the level of the sinuses and was dilated. The descending aorta had an intra-aortic balloon pump, and the right atrium showed a venous line in the right atrium from the inferior vena cava (IVC) to the right ventricle. The follow-up EKG in the clinic after a month showed deep Q waves with T-wave inversion, as in Figure 3.

## Discussion

Cardiac troponin I (cTnI) and T (cTnT) are regulatory proteins (cardiac isoforms) that mediate the calcium-mediated interaction of actin and myosin in the heart muscle [4]. These proteins result from specific genes and are therefore unique to cardiac tissue. Studies with cTnI have not shown to find any cTnI besides the cardiac tissue at any period of neonatal development. In contrast, cTnT is released from skeletal muscle as well. The study indicates that some patients with chronic skeletal muscle diseases express these proteins [5]. The study concludes that these skeletal muscles can be a source of increased cTnT [5] in some patients.

Cardiac troponin (cTn) measurements are enzyme-linked immunosorbent assays in which an antibody captures the material and then a tagged antibody labels it. In most assays, the capture antibodies are monoclonal and specific for the measured troponin, either cardiac troponin T or cardiac troponin I. To increase the amount of captured protein, often two antibodies are used. Each assay is different because the antibodies used in the assays are different. Because of different detection methods and differences in calibration, the values from one assay need to be harmonized with those from other assays [3]. So, any value from one assay cannot substitute for another.

There are many causes for false-negative and false-positive troponin values, as shown in Table 2 [6]. Our discussion will be more focused on false-negative causes [7]. Pronounced hemolysis (>1 g hemoglobin/L) can cause a falsely low cTn [8]. Another cause of false-negative troponin is the excessive use of biotin, a water-soluble B-complex vitamin [9]. It is available as an over-the-counter dietary supplement (5-10 mg) for strengthening hair and nails or as medicine for treating peripheral neuropathy. Very high concentrations of bilirubin (>10 mg/dL) can sometimes interfere with cTnI measurements, resulting in false-negative troponin [8]. High lipid and protein concentrations can also interfere with automated assays. They can also prevent proper sample aspiration, leading to inadequate sample volume. They also cause volume displacement by inhabiting a more significant percentage of plasma volume [10].

**Table 2:** Causes of false negative and positive troponin values.

False negative troponin	False positive troponin
Analysis malfunction	Analysis malfunction
Hyperbilirubinemia	Hemolysis
Lipemia	Heterophile antibodies
Biotin consumption	Fibrin interference
Hemolysis	Elevated alkaline phosphatase
Cardiac troponin autoantibodies	Rheumatoid factor

Regarding our case, analyzer malfunction is less likely [2,4,11]. Repeated troponin measurements during the hospital stay were negative. Troponin sent to the outside lab was also negative. CK-MB was also negative. His bilirubin was never higher than 5.4 mg/dl, and there were no signs of intravascular or extravascular hemolysis. His total lipid level, as well as his protein level, were also normal. He had no history of using over-the-counter vitamins or any other medicine. We could not find the cause of false negative troponin in our patient.

Our patient might have had ACS a month ago, resulting in the development of an LV thrombus, which might eventually have become the source of the emboli occluding the LAD. It is also possible that the LAD occlusion remained critically stenotic for a month and then completely occluded two days before the current presentation.

## Conclusions

This case presents a rare and atypical presentation of negative troponin in a patient with STEMI. With a sensitivity of more than 99%, cardiac troponin is a reliable marker of cardiac injury. Still, it might be negative during the initial phase of acute coronary syndrome and may eventually normalize. It is still unknown why our patient tested negative for troponin despite multiple tests. Physicians should be aware of this rare finding.
